# Post-Marketing Reporting Patterns of Ceftazidime–Avibactam and Ceftolozane–Tazobactam: Analysis of Adverse Event Reports from EudraVigilance

**DOI:** 10.3390/antibiotics15100982

**Published:** 2026-10-05

**Authors:** Viviana Maria Gianguzzo, Michelangelo Rottura, Federica Maria Sacco, Giuseppina Sanna, Matthew Gavino Donadu, Natasha Irrera, Egidio Imbalzano, Giovanni Pallio, Vincenzo Arcoraci

**Affiliations:** 1 Department of Chemical, Biological, Pharmaceutical and Environmental Sciences, University of Messina, 98166 Messina, Italy; vgianguzzo@unime.it; 2 Department of Clinical and Experimental Medicine, University of Messina, 98125 Messina, Italy; fsacco@unime.it (F.M.S.); nirrera@unime.it (N.I.); eimbalzano@unime.it (E.I.); varcoraci@unime.it (V.A.); 3 Department of Biomedical Sciences, University of Cagliari, 09042 Monserrato, Italy; g.sanna@unica.it; 4 Department of Medicine, Surgery and Pharmacy, School of Specialization in Hospital Pharmacy, University of Sassari, 07100 Sassari, Italy; 5 Hospital Pharmacy, Giovanni Paolo II Hospital, ASL Gallura, 07026 Olbia, Italy; 6 Department of Biomedical and Dental Sciences and Morphological and Functional Imaging, University of Messina, 98125 Messina, Italy; gpallio@unime.it

**Keywords:** ceftazidime/avibactam, ceftolozane/tazobactam, EudraVigilance, pharmacovigilance, adverse drug reactions

## Abstract

**Background/Objectives**: Ceftazidime/avibactam (CA) and ceftolozane/tazobactam (CT) are WHO Reserve-group antibiotics increasingly used for multidrug-resistant Gram-negative infections. Comparative post-marketing pharmacovigilance data remain limited. **Methods**: A retrospective pharmacovigilance study was conducted using Individual Case Safety Reports (ICSRs) retrieved from the EudraVigilance database. Reports listing CA or CT as suspected drugs were included. Comparative reporting analyses were performed using reporting odds ratios (RORs) with 95% confidence intervals (CIs) at System Organ Class (SOC) and Preferred Term (PT) levels. **Results**: A total of 1414 ICSRs were identified (CA: 967; CT: 447). Serious reports were more frequent for CA than CT (91.0% vs. 78.7%), whereas fatal outcomes were proportionally higher for CT (21.0% vs. 12.8%). At the SOC level, CA showed higher reporting for hepatobiliary disorders (ROR 2.00; 95% CI 1.20–3.35), investigations (ROR 2.67; 95% CI 1.92–3.71), nervous system disorders (ROR 1.51; 95% CI 1.07–2.15), and psychiatric disorders (ROR 3.25; 95% CI 1.37–7.72). At the PT level, CA showed higher reporting for encephalopathy (ROR 4.25; 95% CI 1.28–14.09), blood creatinine increased (ROR 7.37; 95% CI 1.76–30.93), platelet count decreased (ROR 26.9; 95% CI 3.71–195.0), and diarrhoea (ROR 2.83; 95% CI 1.09–7.34). Death was less frequently reported for CA relative to CT (ROR 0.17; 95% CI 0.10–0.29). **Conclusions**: This EudraVigilance-based analysis identified distinct reporting patterns for CA and CT. CA showed higher reporting for neurological, hepatobiliary, and laboratory-related events, whereas CT reports more frequently involved infection-related contexts. These findings support continued post-marketing surveillance, while clinical monitoring should follow established clinical guidance and product information.

## 1. Introduction

Antimicrobial resistance (AMR) represents one of the most urgent global health challenges, undermining the effectiveness of available therapies and contributing substantially to morbidity, mortality, and healthcare costs worldwide [1]. The progressive dissemination of multidrug-resistant (MDR) Gram-negative pathogens has intensified the need for coordinated strategies aimed at preventing infection, optimizing antibiotic use, and limiting the spread of resistant microorganisms [2]. Combating AMR requires not only the development of novel antimicrobial agents but also the implementation of robust infection prevention and control measures, including hygiene reinforcement, screening of high-risk patients, isolation procedures, environmental decontamination, and structured antimicrobial stewardship programs (ASPs) [2].

To guide antibiotic use globally, the World Health Organization (WHO) introduced the AWaRe classification, categorizing agents into Access, Watch, and Reserve groups [3]. While Access antibiotics are recommended as first-line options for common infections and Watch antibiotics require careful monitoring, Reserve antibiotics are considered last-resort agents intended for infections caused by MDR organisms [4]. Several recently developed cephalosporins and β-lactam/β-lactamase inhibitor (BL/BLI) combinations have been included within the Reserve category because of their crucial role in addressing therapeutic gaps against resistant Gram-negative bacteria [5,6,7].

Among these agents, ceftolozane/tazobactam (CT) and ceftazidime/avibactam (CA) have gained particular relevance in the management of severe infections caused by resistant Gram-negative pathogens. In the European Union (EU), CT received a centralized marketing authorization valid throughout the EU on 18 September 2015, while CA was authorized on 23 June 2016 [8,9]. These dates provide a common European regulatory reference for the availability of the two agents, although their actual market availability, reimbursement status, formulary inclusion, and conditions of clinical access may differ among individual countries and healthcare systems. According to their European Summary of Product Characteristics (SmPC), CT is approved for the treatment of complicated intra-abdominal infections (in combination with metronidazole when anaerobic coverage is required), complicated urinary tract infections including acute pyelonephritis, and hospital-acquired pneumonia including ventilator-associated pneumonia [8]. Similarly, CA is indicated for complicated intra-abdominal infections (in combination with metronidazole), complicated urinary tract infections including pyelonephritis, hospital-acquired pneumonia including ventilator-associated pneumonia, and infections due to aerobic Gram-negative organisms in adults with limited treatment options [9].

From a microbiological perspective, CT combines a potent antipseudomonal cephalosporin with the β-lactamase inhibitor tazobactam, enhancing activity against Pseudomonas aeruginosa and selected extended-spectrum β-lactamase (ESBL)-producing Enterobacterales [5,6]. In contrast, CA consists of a third-generation cephalosporin and the non-β-lactam β-lactamase inhibitor avibactam, which inhibits class A enzymes including KPC and selected class C and D β-lactamases, thereby extending activity against carbapenem-resistant Enterobacterales [5,6]. These mechanistic differences underpin their complementary clinical positioning within antimicrobial stewardship frameworks.

Despite these differences, CA and CT share several clinically relevant features: both are newer cephalosporin/β-lactamase inhibitor combinations classified within the WHO Reserve group, have partially overlapping approved indications, and are used in the management of severe infections caused by resistant Gram-negative pathogens [4,5,6,8,9]. Furthermore, the two agents have previously been evaluated within the same clinical and pharmacovigilance studies [10,11], providing a relevant basis for their comparative evaluation in the post-marketing setting. Evidence from randomized clinical trials has provided information on the efficacy and tolerability of these agents. In the pivotal ASPECT-cIAI (Assessment of the Safety Profile and Efficacy of Ceftolozane/Tazobactam in complicated Intra-Abdominal Infections) phase 3 study, CT plus metronidazole was non-inferior to meropenem in adults with complicated intra-abdominal infections and showed a generally comparable incidence of adverse events, with adverse events reported in 44.0% of treated patients and most events classified as mild to moderate [12]. The most frequently reported adverse reactions were gastrointestinal disorders such as nausea and diarrhoea, as well as pyrexia and headache. Laboratory abnormalities most commonly involved elevations in hepatic transaminases [12]. Real-world cohort data further support the clinical effectiveness and acceptable tolerability of both CT and CA in severe infections, including hospital-acquired pneumonia and bloodstream infections [13,14].

Although pre-authorization data provide important information on adverse reactions, rare or delayed adverse drug reactions (ADRs) may not be fully captured during clinical development. A pharmacovigilance analysis based on the US FDA Adverse Event Reporting System (FAERS) identified reporting signals of interest for both agents, emphasizing the importance of ongoing post-marketing surveillance [11]. Within Europe, EudraVigilance (EV), developed by the European Medicines Agency, provides a centralized system for the collection and evaluation of suspected ADRs [15]. EV-based analyses enable the descriptive characterization of Individual Case Safety Reports (ICSRs) and the application of disproportionality methods, such as reporting odds ratios (RORs), to explore potential pharmacovigilance signals [16,17]. Although spontaneous reporting systems are inherently subject to under-reporting and reporting bias, they remain valuable tools for identifying emerging reporting patterns and potential pharmacovigilance signals [18], particularly with regard to antibiotics reserved for severe infections.

Given the strategic importance of CT and CA within the WHO Reserve group, further characterization of their post-marketing reporting patterns using European pharmacovigilance data is warranted. Although individual descriptive characteristics of ICSRs are publicly accessible through EV, their systematic comparative evaluation may provide relevant context for interpreting differences in reporting patterns between the two agents. Therefore, the aim of the present study was to characterize and compare suspected adverse drug reactions reported for these two emerging antimicrobials in the EV database over their respective post-authorization periods.

## 2. Results

### 2.1. Descriptive Analysis of ICSRs

A total of 1414 ICSRs were identified in the EudraVigilance database, including 967 reports for CA and 447 reports for ceftolozane/tazobactam.

Male patients were more frequently represented in both groups (61.4% for CA and 55.3% for CT), whereas females accounted for 32.0% and 34.7% of reports, respectively (*p* = 0.017). The most represented age category was 18–64 years (36.6% for CA and 33.3% for CT), followed by patients aged 65–85 years (33.0% vs. 26.4%). A significantly higher proportion of reports with unspecified age was observed for CT compared with CA (34.0% vs. 15.5%; *p* < 0.001). Overall, age distribution differed significantly between the two drugs (*p* < 0.001).

Most ICSRs were classified as serious, with a significantly higher proportion observed for CA than CT (91.0% vs. 78.8%; *p* < 0.001). Regarding outcomes, fatal cases were proportionally more frequent among CT reports (21.0% vs. 12.8%), whereas recovering/resolving outcomes were more commonly reported for CA (28.7% vs. 12.5%) (*p* < 0.001).

The majority of reports originated from healthcare professionals, particularly for CT (98.2% vs. 85.3% for CA; *p* < 0.001). Regarding geographical origin, reports from the European Economic Area (EEA) were more frequently observed for CT (58.6%), whereas CA reports predominantly originated from non-EEA countries (61.1%) (*p* < 0.001) (Table 1).

### 2.2. Distribution of Reported Suspected Adverse Reactions by System Organ

A total of 3251 suspected adverse reactions were identified across the retrieved ICSRs, corresponding to a mean of 2.3 reactions per ICSR. Specifically, 2156 suspected adverse reactions were reported in CA-related ICSRs, with a mean of 2.2 reactions per ICSR, whereas 1095 suspected adverse reactions were reported in CT-related ICSRs, corresponding to a mean of 2.4 reactions per ICSR.

For CA, the most frequently reported System Organ Classes (SOCs) were investigations (14.4%), general disorders and administration site conditions (13.3%), skin and subcutaneous tissue disorders (9.7%), injury, poisoning and procedural complications (9.1%), and infections and infestations (8.9%).

For CT, the most frequent SOCs included general disorders and administration site conditions (17.9%), injury, poisoning and procedural complications (16.6%), infections and infestations (11.3%), blood and lymphatic system disorders (6.2%), and renal and urinary disorders (5.6%).

The comparative ROR analysis at the SOC level identified differences in reporting patterns between CA and CT. In particular, CA showed significantly higher reporting for hepatobiliary disorders (ROR 2.00; 95% CI 1.20–3.35) and investigations (ROR 2.67; 95% CI 1.92–3.71). Higher reporting frequency was observed for nervous system disorders (ROR 1.51; 95% CI 1.07–2.15) and psychiatric disorders (ROR 3.25; 95% CI 1.37–7.72), as well as for skin and subcutaneous tissue disorders (ROR 1.39; 95% CI 1.01–1.93).

Conversely, CA showed significantly lower reporting for general disorders and administration site conditions (ROR 0.59; 95% CI 0.46–0.75), infections and infestations (ROR 0.67; 95% CI 0.50–0.89), and injury, poisoning and procedural complications (ROR 0.41; 95% CI 0.31–0.53), suggesting a relatively higher representation of these SOCs among CT reports. No statistically significant differences were observed for renal and urinary disorders (ROR 0.99; 95% CI 0.69–1.43) (Table 2).

### 2.3. Comparative ROR Analysis at Preferred Term Level

At the Preferred Term (PT) level, several statistically significant differences in reporting were observed. Among the most notable findings, CA showed a markedly higher reporting for hematological and laboratory-related PTs, including decreased platelet count (ROR 26.9; 95% CI 3.71–195.0) and decreased white blood cell count (ROR 4.94; 95% CI 1.15–21.16). Laboratory abnormalities were further supported by the increased reporting of blood creatinine levels (ROR 7.37; 95% CI 1.76–30.93).

Neurological and neuropsychiatric PTs also showed differences in reporting patterns. Encephalopathy showed significantly higher reporting for CA (ROR 4.25; 95% CI 1.28–14.09), whereas other neurological and neuropsychiatric PTs, including delirium and altered state of consciousness, showed RORs above 1 but with wide 95% CIs encompassing unity.

Among gastrointestinal PTs, diarrhoea also showed significantly higher reporting for CA (ROR 2.83; 95% CI 1.09–7.34).

In contrast, several PTs showed significantly lower reporting for CA, including death (ROR 0.17; 95% CI 0.10–0.29), product use in unapproved indication (ROR 0.08; 95% CI 0.04–0.16), and Pseudomonas infection (ROR 0.24; 95% CI 0.10–0.57). Additionally, no statistically significant differences were observed for renal-related PTs, including renal impairment, acute kidney injury, and renal failure (Table 3).

In the age-stratified analysis, nervous system disorders showed a numerically higher ROR for CA versus CT in patients aged ≥65 years (ROR 1.83, 95% CI 1.00–3.36) than in those aged 18–64 years (ROR 1.05, 95% CI 0.61–1.81). At the PT level, none of the neurological or neuropsychiatric PTs showed statistically significant higher reporting in either age group. Among the other PTs, platelet count decreased showed significantly higher reporting in patients aged ≥65 years (ROR 11.54; 95% CI 1.56–85.09). Complete results of the age-stratified analyses are reported in Supplementary Tables S1 and S2.

The sensitivity analysis restricted to serious ICSRs included 1232 reports, comprising 880 ICSRs for CA and 352 for CT. Overall, the main reporting patterns observed in the primary analysis were maintained. At the SOC level, significantly higher reporting for CA remained for hepatobiliary disorders (ROR 1.81; 95% CI 1.05–3.11), investigations (ROR 2.94; 95% CI 2.03–4.27), nervous system disorders (ROR 1.51; 95% CI 1.04–2.19), psychiatric disorders (ROR 4.14; 95% CI 1.47–11.67), and skin and subcutaneous tissue disorders (ROR 1.64; 95% CI 1.06–2.53). Conversely, the lower reporting observed for several SOCs in the primary analysis, including general disorders and administration site conditions, infections and infestations, and injury, poisoning and procedural complications, was also maintained.

At the PT level, the principal findings of the primary analysis were also maintained after restriction to serious ICSRs. In particular, platelet count decreased (ROR 21.59; 95% CI 2.97–156.87), blood creatinine increased (ROR 12.82; 95% CI 1.74–94.25), and encephalopathy (ROR 3.68; 95% CI 1.11–12.22) continued to show significantly higher reporting for CA. Estimates for several other neurological and neuropsychiatric PTs remained imprecise, with wide confidence intervals encompassing unity. Complete results of the sensitivity analysis are reported in Supplementary Tables S3 and S4.

Comparison with the respective European SmPCs showed that several of the PTs identified in EV were already represented as listed adverse reactions or clinically corresponding adverse reactions in the product information. For CA, some neurological PTs identified in the present analysis were not listed as adverse reactions in Section 4.8 of the Zavicefta SmPC but were mentioned elsewhere in the product information in the context of neurological manifestations. The complete PT-level comparison with the respective European SmPCs is provided in Supplementary Table S5.

## 3. Discussion

This comparative pharmacovigilance study based on EV data revealed distinct reporting patterns for CA and CT. Overall, CA accounted for a higher number of ICSRs and showed higher reporting for selected neurological, psychiatric, hepatobiliary, and laboratory-related events. In contrast, CT reports were more frequently associated with infection-related and procedural contexts. These findings should be interpreted cautiously because comparative ROR analyses performed in spontaneous reporting systems reflect differences in reporting rather than causal associations, incidence, or comparative risk [19,20,21].

At the SOC level, CA showed higher reporting for nervous system disorders, psychiatric disorders, hepatobiliary disorders, investigations, and skin and subcutaneous tissue disorders. Conversely, CT reports more frequently involved infections and infestations, injury/procedural complications, and general disorders. These differences may partially reflect the heterogeneous clinical settings in which these agents are commonly prescribed. Real-world studies evaluating CT have documented frequent use in critically ill patients with severe multidrug-resistant *Pseudomonas aeruginosa* infections, including ventilator-associated pneumonia and bloodstream infections [14]. Therefore, the increased representation of infection- and procedure-related SOCs among CT reports may plausibly reflect the complexity of the treated population rather than drug-specific adverse reaction patterns.

Among the most clinically relevant findings, CA showed higher reporting for nervous system disorders at the SOC level. At the PT level, encephalopathy showed significantly higher reporting for CA, whereas several other neurological and neuropsychiatric PTs showed RORs above 1 but with wide confidence intervals encompassing unity. These findings appear consistent with the well-described neurotoxicity associated with cephalosporins and other β-lactam antibiotics [22]. Cephalosporin-associated neurotoxicity has been linked to concentration-dependent gamma-aminobutyric acid (GABA) antagonism and excitatory pathway dysregulation, while advanced age, renal impairment, excessive dosage, and critical illness have consistently been identified as major predisposing factors [22].

In the age-stratified analysis, none of the neurological or neuropsychiatric events showed statistically significant higher reporting within either age group. Advanced age has previously been recognized as a potential predisposing factor for β-lactam-associated neurotoxicity, particularly in the presence of renal impairment, and pharmacovigilance data have specifically raised attention to neurological events associated with CA in patients older than 65 years [23]. Therefore, our findings do not provide evidence of a specific age-related reporting pattern and should be interpreted cautiously, particularly considering the limited number of reports and the wide confidence intervals for individual PTs.

The sensitivity analysis restricted to serious ICSRs showed that the main reporting patterns were generally maintained.

Despite restricting the analysis to 1232 serious reports, the principal SOC-level findings were generally maintained, including higher reporting for hepatobiliary disorders, investigations, nervous system disorders, psychiatric disorders, and skin and subcutaneous tissue disorders with CA. At the PT level, platelet count decreased, blood creatinine increased, and encephalopathy also remained significantly more frequently reported for CA. These findings suggest that the principal reporting differences observed in the primary analysis were not solely driven by the different proportion of serious reports between CA and CT. Nevertheless, restriction to serious ICSRs does not eliminate other sources of confounding or reporting bias inherent to spontaneous reporting data.

The potential role of renal dysfunction in the observed neurological reporting patterns deserves particular attention. The comparison with the European product information further contextualized these findings. Some neurological PTs observed for CA, including encephalopathy, seizure-related events, myoclonus, tremor, and coma, are not listed as adverse reactions in Section 4.8 of the Zavicefta SmPC but are mentioned elsewhere in the product information in the context of neurological manifestations, particularly when the ceftazidime dose has not been appropriately reduced in patients with renal impairment [9]. In patients with chronic kidney disease, inadequate dose adjustment of β-lactam antibiotics may result in drug accumulation and increased toxicity [24]. Consistent with established clinical knowledge and the recommendations provided in the product information, both CA and CT require dose adjustment in patients with impaired renal function, and renal function should be assessed before treatment and monitored during therapy, particularly when it is rapidly changing. In vulnerable patients receiving CA, especially older adults and patients with renal impairment, careful monitoring of renal function and neurological status is particularly relevant [8,9,24]. For CT, renal function should likewise be monitored during treatment and the dose adjusted according to creatinine clearance, particularly when renal function changes during therapy [8]. Advanced age alone does not require dose adjustment for either agent; rather, dosing in older patients should be guided by renal function [8,22]. In critically ill patients, in whom renal function may fluctuate considerably during the course of infection, repeated assessment of renal function and corresponding reassessment of the dosing regimen may be particularly important [10]. Additional support for the concentration-dependent nature of cephalosporin neurotoxicity derives from studies evaluating cefepime-induced neurotoxicity. A systematic review focused on cefepime-induced neurotoxicity reported that most cases occurred in subjects with renal impairment and frequently involved altered mental status, myoclonus, and non-convulsive seizures [25]. Although cefepime differs structurally from CA and CT, these findings reinforce the broader concept of concentration-dependent neurotoxicity among antipseudomonal cephalosporins. Consistent with this established clinical evidence, appropriate renal dose adjustment and careful neurological monitoring remain important in susceptible patients [25].

At the PT level, CA also showed higher reporting of laboratory abnormalities, including increased blood creatinine and decreased platelet count. Notably, both findings remained statistically significant in the sensitivity analysis restricted to serious ICSRs, although the estimates remained imprecise, as reflected by the wide confidence intervals.

The comparison with the European product information further showed that these findings are represented in the CA SmPC, with increased blood creatinine listed as an adverse reaction and decreased platelet count clinically corresponding to the listed adverse reaction thrombocytopenia [9] (Supplementary Table S5).

These findings should be interpreted with caution, particularly for PTs characterized by relatively low frequencies and wide confidence intervals. Nevertheless, partially overlapping findings have been reported in previous pharmacovigilance studies. A large-scale FAERS analysis evaluating novel β-lactam/β-lactamase inhibitor combinations identified neurological and hematological events among the most relevant disproportionality signals reported for CA and CT [11]. Although direct comparison between FAERS and EV should be approached cautiously because of methodological and regulatory differences between pharmacovigilance databases [14,19,20,21], the partial consistency of reporting patterns across different systems may support continued pharmacovigilance surveillance of these.

Conversely, the PT death, product use in unapproved indication, and Pseudomonas infection were less frequently reported for CA relative to CT. These findings should not be interpreted as evidence of differential mortality risk or comparative safety between the two agents. In spontaneous reporting systems, fatal outcomes may be influenced by several confounding factors, including infection severity, intensive care admission, microbiological characteristics, baseline comorbidities, and concomitant therapies [14,20,21]. Comparative cohort studies evaluating CA and CT in multidrug-resistant Gram-negative infections did not identify substantial differences in major clinical outcomes between the two agents [26].

The present findings should also be interpreted within the broader context of antimicrobial stewardship. Both CA and CT belong to the WHO Reserve group and are typically prescribed for difficult-to-treat multidrug-resistant Gram-negative infections [4]. Previous EV-based analyses involving Reserve cephalosporins highlighted the potential role of pharmacovigilance approaches in identifying reporting patterns related not only to adverse drug reactions, but also to resistance, ineffectiveness, and off-label use [6]. The present analysis should be viewed as complementary to the recent EudraVigilance study by Arseniu et al. [6], which focused specifically on reporting signals related to resistance, ineffectiveness, and off-label use across Reserve-group cephalosporins. In contrast, the present study provides a focused comparison of CA and CT across a broader spectrum of suspected adverse reactions at both SOC and PT levels, complemented by age-stratified analyses and a sensitivity analysis restricted to serious ICSRs. In this context, spontaneous reporting systems may provide complementary post-marketing information useful for stewardship-oriented surveillance of recently introduced antimicrobials [20].

Compared with pre-authorization evidence, the present findings complement rather than contradict available clinical trial data. In the ASPECT-cIAI phase 3 trial, CT plus metronidazole showed tolerability findings generally comparable to those observed with meropenem, with gastrointestinal disorders and laboratory abnormalities among the most frequently reported adverse reactions [12]. Similarly, observational studies evaluating CA in routine clinical practice have generally described acceptable tolerability profiles in severe infections [13]. However, randomized clinical trials and observational cohorts may be limited in their ability to identify uncommon, delayed, or context-dependent adverse reactions, particularly in highly heterogeneous and critically ill populations. Therefore, post-marketing pharmacovigilance analyses can provide additional information on the reporting of suspected adverse reactions in routine clinical practice.

## 4. Limitations and Strengths

This study has several limitations inherent to spontaneous reporting systems. First, EudraVigilance data are subject to under-reporting, selective and stimulated reporting, incomplete clinical information, variable coding practices, and potential residual duplicate reporting [19,20,21]. Although EudraVigilance applies centralized procedures for duplicate detection and management and ICSRs were identified in the present analysis by their EULocalNumber, residual duplication cannot be completely excluded. Furthermore, the absence of exposure denominators prevents estimation of incidence or absolute risk, and the comparative RORs used in this study reflect differences in reporting between CA and CT rather than causal associations, relative risk, or comparative safety [21]. Accordingly, differences in the absolute number of ICSRs between the two agents should not be interpreted as differences in event frequency or safety.

Second, CA and CT differ in their microbiological spectrum, therapeutic positioning, and patterns of clinical use. Consequently, the underlying pathogens, patient populations, infection severity, treatment settings, and position in the therapeutic pathway may differ between the two agents, introducing potential confounding by indication and clinical severity. Although information on therapeutic indication, route of administration, treatment duration, dosage regimen, and concomitant medications was retrieved when available, these variables were insufficiently complete and homogeneous at the individual-case level to permit reliable stratified or multivariable-adjusted analyses. Similarly, information on renal function, renal dose adjustment, treatment start and event-onset dates, concomitant nephrotoxic or neurotoxic medications, and alternative etiologies was unavailable or insufficiently complete for systematic adjustment. Therefore, the observed reporting differences cannot be attributed independently to the suspected medicinal products.

Third, differences in market availability, reimbursement, formulary inclusion, antimicrobial stewardship policies, prescribing practices, and conditions of clinical access across countries and healthcare systems may influence both drug utilization and spontaneous reporting. Because geographical information in the present analysis was available only according to EEA versus non-EEA origin, country-level differences in access, utilization, or reporting practices could not be evaluated. Therefore, the observed geographical distribution of reports should not be attributed to specific national or regional policies. Differences in case counts between this study and previous EudraVigilance analyses may likewise reflect differences in study periods, eligibility criteria, report sources, extraction procedures, and case-selection or data-processing procedures and should therefore be interpreted cautiously.

Fourth, the SOC- and PT-level comparative analyses involved multiple statistical comparisons, and no formal adjustment for multiple testing was applied because the analyses were exploratory and intended for hypothesis generation rather than confirmatory inference. The 23 SOC-level and 92 PT-level comparisons referred to the primary analysis; additional comparisons were performed in the age-stratified and serious-ICSR sensitivity analyses, for which no formal multiplicity adjustment was likewise applied. Although a minimum reporting threshold was used in the primary analysis to reduce the influence of extremely sparse data, this criterion does not eliminate statistical instability. Estimates based on small numbers of ICSRs or characterized by wide confidence intervals should therefore be interpreted cautiously. In addition, multiple PTs may be reported within the same ICSR, and clinically related PTs may represent different manifestations of the same underlying clinical condition. Consequently, individual PT-level findings should not be regarded as completely independent pharmacovigilance signals.

Despite these limitations, this study has several strengths. It provides a focused head-to-head characterization of spontaneous reporting patterns for two clinically related WHO Reserve cephalosporin/β-lactamase inhibitor combinations, using the ICSR rather than the individual reaction record as the unit of analysis. Reporting patterns were evaluated at both SOC and PT levels using a consistent comparative ROR framework, with CT serving as an active comparator rather than the entire EudraVigilance database. The primary analysis was complemented by an age-stratified comparative ROR analysis and a sensitivity analysis restricted to serious ICSRs, with the latter showing that the principal reporting patterns were generally maintained despite the different proportions of serious reports between the two drugs. Selected PT-level findings were also examined descriptively at the individual-case level, where the available information permitted further clinical contextualization. Finally, the systematic comparison of PTs with the respective European SmPCs provides additional regulatory and clinical context by distinguishing findings already represented in the European product information from those not specifically listed as adverse reactions. Taken together, these complementary approaches strengthen the descriptive and hypothesis-generating value of the analysis while maintaining the limitations inherent to spontaneous reporting data.

## 5. Materials and Methods

### 5.1. Data Source and Study Design

A retrospective pharmacovigilance study was performed using data extracted from the publicly accessible EV database (European Medicines Agency; available at EudraVigilance public access portal www.adrreports.eu; accessed on 14 February 2026). EV represents the European Union system dedicated to the collection and management of spontaneous reports of suspected adverse drug reactions (ADRs) associated with medicinal products authorized within the European Economic Area, as well as investigational products evaluated in clinical studies. The database is widely employed for post-marketing pharmacovigilance and pharmacoepidemiological investigations, providing information on spontaneously reported suspected adverse reactions to medicinal products. Previous investigations have highlighted its utility in signal detection and characterization of potential pharmacovigilance signals.

### 5.2. Identification and Selection of Safety Reports

ICSRs reporting CT or CA as suspected medicinal products were initially retrieved from EV. In the initial extraction, the earliest retrieved ICSR was dated 2 June 2015 for CT and 17 October 2015 for CA, while the most recent reports were dated 12 February 2026 and 6 February 2026, respectively. These dates refer to the initial unfiltered extraction and do not define the final post-marketing analytical dataset. The eligibility criteria described below were subsequently applied to identify the ICSRs included in the analysis. Data extraction was completed on 14 February 2026.

Only spontaneous post-marketing ICSRs reporting CT or CA as suspected medicinal products were included. Reports originating exclusively from scientific literature were excluded. EV applies centralized procedures for duplicate detection and management, with confirmed duplicates consolidated into master cases and follow-up reports managed as updates to the same individual case rather than as separate ICSRs. In the present analysis, ICSRs were identified by their EULocalNumber, and multiple reaction records sharing the same identifier were counted as a single ICSR.

### 5.3. Variables and Data Processing

For each eligible ICSR, the following information was collected: EV identification code, reporting date, and qualification of the primary notifier. Additional variables included patient-related characteristics (sex and age category), as well as ADR-related information such as reaction type, seriousness, clinical outcome, and duration when available. Data concerning suspected and concomitant medicinal products were also retrieved, including therapeutic indication, route of administration, treatment duration, and dosage regimen.

All ADRs were classified according to the Medical Dictionary for Regulatory Activities (MedDRA^®^), version 28.1. MedDRA terminology follows a hierarchical structure composed of five levels: Lowest Level Terms (LLTs), PTs, High-Level Terms (HLTs), High-Level Group Terms (HLGTs), and SOCs.

Preferred Terms reported in at least three ICSRs for either CA or CT were additionally compared with the adverse reactions listed in the respective SmPC. A PT was considered listed when either the same term or a clinically corresponding adverse reaction was reported in the SmPC; PTs mentioned elsewhere in the product information but not listed as adverse reactions were identified separately.

Reactions were considered serious when at least one of the following regulatory seriousness criteria was documented in the ICSR: fatal outcome, life-threatening condition, hospitalization or prolonged hospitalization, persistent or clinically relevant disability/incapacity, congenital anomaly, or other medically important condition. Clinical outcomes were categorized according to the standardized EV terminology as follows: recovered/resolved, recovering/resolving, recovered/resolved with sequelae, not recovered/not resolved, fatal, and unknown.

Medicinal products were categorized using the Anatomical Therapeutic Chemical (ATC) classification system. In cases where multiple ADRs within the same ICSR were associated with different clinical outcomes, the overall case outcome was assigned according to the lowest level of resolution approach previously described in pharmacovigilance studies.

### 5.4. Statistical Analysis

A descriptive statistical approach was adopted to characterize ICSRs, patient demographics, and ADR distributions. Categorical variables were expressed as frequencies and percentages. Comparative analyses between the two antibiotic groups were performed using Pearson’s chi-square test or an exact test, as appropriate according to expected cell frequencies. All tests were two-tailed, and a *p*-value < 0.05 was considered statistically significant.

CT was used as an active comparator for CA because the two agents belong to the same broad therapeutic class and share partially overlapping clinical indications. However, given their different microbiological spectra and therapeutic positioning, the comparison was designed to investigate differences in reporting patterns rather than to estimate relative toxicity or comparative safety.

Comparative ROR analyses were subsequently performed to quantify differences in reporting patterns between CA and CT, with CT serving as the active comparator. The ICSR was used as the unit of analysis. For each SOC or PT, an ICSR was counted once when at least one corresponding reaction was reported, irrespective of the number of reactions belonging to the same SOC or the repetition of the same PT within that report. For each SOC or PT, a 2 × 2 contingency table was constructed, where *a* represented CA ICSRs containing the event of interest, *b* CA ICSRs not containing the event, *c* CT ICSRs containing the event, and *d* CT ICSRs not containing the event. The ROR was calculated as (*a/b*)/(*c/d*) = *ad/bc*.

RORs and corresponding 95% confidence intervals (95% CIs) were calculated at both the SOC and PT levels according to MedDRA classification. Higher reporting of a given SOC or PT for CA relative to CT was considered statistically significant when the lower bound of the 95% CI was greater than 1, whereas lower reporting was considered statistically significant when the upper bound of the 95% CI was below 1. Only events reported in at least three ICSRs were included in the primary comparative ROR analysis, which comprised 23 SOC-level and 92 PT-level comparisons. No formal adjustment for multiple testing was applied to either the primary analysis or the additional age-stratified and sensitivity analyses, as these analyses were exploratory and intended for hypothesis generation rather than confirmatory inference. RORs were interpreted as measures of differential reporting between CA and CT and not as estimates of incidence, relative risk, causality, or comparative safety.

An age-stratified comparative ROR analysis was performed separately in patients aged 18–64 years and ≥65 years. The original age categories of 65–85 years and >85 years were combined into a single ≥65-year group to provide a clinically meaningful older-adult stratum and increase the number of observations. Pediatric age categories (0–1 month, 2 months–2 years, 3–11 years, and 12–17 years) were excluded because of the limited number of reports and were not pooled due to the clinical and physiological heterogeneity across developmental stages. Reports with unspecified age were also excluded. Within each age stratum, RORs and corresponding 95% CIs were calculated using the same ICSR-based comparative approach applied in the primary analysis. The analysis was performed at both the SOC and PT levels, with particular attention to neurological and neuropsychiatric PTs.

As a sensitivity analysis, the comparative ROR analysis was repeated after restricting the dataset to serious ICSRs. This analysis was performed to assess whether the main reporting patterns observed in the primary analysis were robust to differences in the distribution of report seriousness between CA and CT. The same ICSR-based approach used in the primary analysis was applied at both the SOC and PT levels. To preserve direct comparability with the primary analysis, the sensitivity analysis was restricted to the SOCs and PTs that met the eligibility criterion in the primary dataset; the ≥3-ICSR threshold was not reapplied within the serious-report subset. When an event was not reported for one of the two drugs in the serious subset, the conventional ROR was considered not estimable.

All analyses were performed using IBM SPSS Statistics, version 23.0 (IBM Corp., Armonk, NY, USA).

## 6. Conclusions

This EV-based pharmacovigilance analysis revealed different reporting patterns for CA and CT. CA showed higher reporting for neurological, psychiatric, hepatobiliary, and selected laboratory-related PTs, whereas CT reports more frequently involved infection-related and procedural contexts. Although these findings do not establish causality, they highlight the importance of ongoing post-marketing surveillance.

Clinical monitoring, particularly in patients with renal dysfunction or other established risk factors for β-lactam neurotoxicity, should be guided by established clinical knowledge and product information rather than inferred from these reporting patterns.

## Figures and Tables

**Table 1:** Characteristics of Individual Case Safety Reports (ICSRs) for ceftazidime/avibactam and ceftolozane/tazobactam.

	Characteristics	CA N 967 (%)	CT N 447 (%)	*p* Value
Sex	Female	309 (32.0)	155 (34.7)	0.017
	Male	594 (61.4)	246 (55.3)	
	Not Specified	64 (6.6)	46 (10.2)	
Age Categories (Years)	0–1 Month	1 (0.1)	-	<0.001
	2 Months–2 Years	12 (1.2)	1 (0.2)	
	3–11 Years	11 (1.1)	4 (0.9)	
	12–17 Years	13 (1.3)	7 (1.6)	
	18–64 Years	354 (36.6)	149 (33.3)	
	65–85 Years	319 (33.0)	118 (26.4)	
	More than 85 Years	107 (11.1)	16 (3.6)	
	Not Specified	150 (15.5)	152 (34.0)	
Seriousness	Non-serious	87 (9.0)	95 (21.3)	<0.001
	Serious	880 (91.0)	352 (78.8)	
Outcome	Fatal	124 (12.8)	94 (21.0)	<0.001
	Not Recovered/Not Resolved	90 (9.3)	40 (9.0)	
	Recovered/Resolved With Sequelae	3 (0.3)	2 (0.5)	
	Recovering/Resolving	277 (28.7)	56 (12.5)	
	Recovered/Resolved	210 (21.7)	118 (26.4)	
	Unknown	263 (27.2)	137 (30.7)	
Primary Source Qualification	Healthcare Professional	825 (85.3)	439 (98.2)	<0.001
	Non-Healthcare Professional	142 (14.7)	8 (1.8)	
Country	European Economic Area	376 (38.9)	262 (58.6)	<0.001
	Non-European Economic Area	591 (61.1)	185 (41.4)	

*p*-values: Pearson’s chi-square test for sex, seriousness, primary source qualification, and country; exact test for age categories and outcome.

**Table 2:** MedDRA^®^ system organ class according to study group ROR: ceftazidime/avibactam versus ceftolozane/tazobactam.

SOC	ROR [95% CI]
Blood and lymphatic system disorders	0.85 [0.59–1.21]
Cardiac disorders	0.78 [0.44–1.37]
Congenital, familial and genetic disorders	0.46 [0.06–3.28]
Ear and labyrinth disorders	1.16 [0.22–5.98]
Eye disorders	2.33 [0.67–8.10]
Gastrointestinal disorders	1.28 [0.82–2.00]
General disorders and administration site conditions	0.59 [0.46–0.75]
Hepatobiliary disorders	2.00 [1.20–3.35]
Immune system disorders	1.24 [0.57–2.69]
Infections and infestations	0.67 [0.50–0.89]
Injury, poisoning and procedural complications	0.41 [0.31–0.53]
Investigations	2.67 [1.92–3.71]
Metabolism and nutrition disorders	0.50 [0.27–0.94]
Musculoskeletal and connective tissue disorders	0.54 [0.24–1.22]
Neoplasms benign, malignant and unspecified (incl cysts and polyps)	0.92 [0.34–2.48]
Nervous system disorders	1.51 [1.07–2.15]
Product issues	0.09 [0.02–0.42]
Psychiatric disorders	3.25 [1.37–7.72]
Renal and urinary disorders	0.99 [0.69–1.43]
Respiratory, thoracic and mediastinal disorders	0.64 [0.38–1.06]
Skin and subcutaneous tissue disorders	1.39 [1.01–1.93]
Surgical and medical procedures	0.12 [0.05–0.30]
Vascular disorders	0.67 [0.37–1.22]

SOC, System Organ Class.

**Table 3:** Comparative ROR analysis at the Preferred Term level for ceftazidime/avibactam versus ceftolozane/tazobactam.

Preferred Terms	CA	ROR [95% CI]
Off-label use	111	0.91 [0.64–1.28]
Drug ineffective	56	1.47 [0.85–2.52]
Platelet count decreased	55	26.9 [3.71–195]
Rash	50	1.57 [0.87–2.83]
Renal impairment	41	1.37 [0.74–2.54]
Pathogen resistance	37	0.57 [0.35–0.95]
Blood creatinine increased	31	7.37 [1.76–30.93]
Erythema	31	1.31 [0.65–2.64]
Diarrhoea	30	2.83 [1.09–7.34]
Pyrexia	29	1.23 [0.61–2.48]
Seizure	29	1.23 [0.61–2.48]
Product use issue	28	1.08 [0.54–2.15]
Thrombocytopenia	28	0.8 [0.43–1.5]
Encephalopathy	27	4.25 [1.28–14.09]
Drug resistance	25	0.96 [0.48–1.93]
Acute kidney injury	23	0.88 [0.44–1.79]
Pruritus	23	1.06 [0.5–2.26]
Condition aggravated	22	2.58 [0.88–7.53]
Renal failure	21	0.69 [0.35–1.36]
White blood cell count decreased	21	4.94 [1.15–21.16]
Death	20	0.17 [0.1–0.29]
Septic shock	19	1.47 [0.58–3.71]
Rash maculo-papular	18	2.1 [0.71–6.24]
Hepatic enzyme increased	16	7.5 [0.99–56.76]
Transaminases increased	16	2.49 [0.72–8.59]
Delirium	15	3.51 [0.8–15.4]
Multiple organ dysfunction syndrome	15	1.16 [0.45–3]
Coma	14	6.55 [0.86–49.98]
Eosinophilia	14	1.63 [0.53–4.97]
Sepsis	14	0.81 [0.34–1.94]
Depressed level of consciousness	13	6.08 [0.79–46.6]
Drug hypersensitivity	13	2.02 [0.57–7.11]
Epilepsy	12	2.8 [0.62–12.54]
Cholestasis	11	1.02 [0.35–2.95]
Dyspnoea	11	1.02 [0.35–2.95]
Hypotension	11	0.85 [0.31–2.3]
Tremor	11	2.56 [0.57–11.6]
Urticaria	11	0.85 [0.31–2.3]
Liver injury	10	4.66 [0.59–36.52]
Myoclonus	10	1.55 [0.42–5.65]
Pneumonia	10	0.51 [0.21–1.26]
Drug eruption	9	4.19 [0.53–33.17]
Infection	9	1.04 [0.32–3.4]
Myelosuppression	9	4.19 [0.53–33.17]
Product use in unapproved indication	9	0.08 [0.04–0.16]
Agranulocytosis	8	0.52 [0.19–1.46]
Altered state of consciousness	8	1.86 [0.39–8.78]
Hepatic cytolysis	8	0.92 [0.28–3.08]
Neutropenia	8	0.46 [0.17–1.23]
Pseudomonas infection	8	0.24 [0.1–0.57]
C-reactive protein increased	7	0.64 [0.2–2.04]
Gastrointestinal haemorrhage	7	0.81 [0.24–2.77]
Hypernatraemia	7	1.08 [0.28–4.19]
Klebsiella infection	7	3.25 [0.4–26.51]
Oxygen saturation decreased	7	3.25 [0.4–26.51]
Respiratory failure	7	0.46 [0.16–1.31]
Anaemia	6	0.46 [0.15–1.43]
Coagulopathy	6	2.78 [0.33–23.2]
Drug level increased	6	2.78 [0.33–23.2]
Nausea	6	2.78 [0.33–23.2]
Urinary tract infection	6	1.39 [0.28–6.91]
Vomiting	6	1.39 [0.28–6.91]
Confusional state	5	1.16 [0.22–5.98]
Face oedema	5	2.32 [0.27–19.9]
Flushing	5	0.77 [0.18–3.23]
Headache	5	0.77 [0.18–3.23]
Incorrect dose administered	5	0.29 [0.09–0.88]
Leukopenia	5	0.33 [0.1–1.04]
Melaena	5	2.32 [0.27–19.9]
Multiple-drug resistance	5	1.16 [0.22–5.98]
Rash pruritic	5	2.32 [0.27–19.9]
Therapeutic product effect incomplete	5	0.77 [0.18–3.23]
White blood cell count increased	5	0.77 [0.18–3.23]
Clostridium difficile colitis	4	0.23 [0.07–0.76]
Clostridium difficile infection	4	0.61 [0.14–2.76]
Dizziness	4	0.92 [0.17–5.06]
Drug interaction	4	1.85 [0.21–16.62]
Drug reaction with eosinophilia and systemic symptoms	4	0.92 [0.17–5.06]
Fungal infection	4	1.85 [0.21–16.62]
Gamma-glutamyltransferase increased	4	1.85 [0.21–16.62]
Generalised tonic–clonic seizure	4	0.61 [0.14–2.76]
Hyperpyrexia	4	1.85 [0.21–16.62]
Hypersensitivity	4	0.92 [0.17–5.06]
Hypokalaemia	4	0.31 [0.09–1.09]
Jaundice	4	1.85 [0.21–16.62]
Malaise	4	0.92 [0.17–5.06]
Muscle twitching	4	1.85 [0.21–16.62]
Overdose	4	0.31 [0.09–1.09]
Pain	4	0.92 [0.17–5.06]
Red blood cell count decreased	4	1.85 [0.21–16.62]
Status epilepticus	4	1.85 [0.21–16.62]
Tachycardia	4	0.92 [0.17–5.06]

CA, ceftazidime/avibactam.

## Data Availability

This study was entirely based on publicly anonymized data made available by the European Medicines Agency. A preprocessed version of the data can be downloaded at the following link: https://www.adrreports.eu/ (accessed on 14 February 2026).

## Supplementary Materials

The following supporting information can be downloaded at https://www.mdpi.com/article/10.3390/antibiotics15100982/s1. Table S1: Age-Stratified Comparative ROR Analysis at the System Organ Class Level for Ceftazidime/Avibactam versus Ceftolozane/Tazobactam; Table S2: Age-Stratified Comparative ROR Analysis at the Preferred Term Level for Ceftazidime/Avibactam versus Ceftolozane/Tazobactam; Table S3: Sensitivity analysis restricted to serious ICSRs: comparative ROR analysis at the System Organ Class level for ceftazidime/avibactam versus ceftolozane/tazobactam; Table S4: Sensitivity analysis restricted to serious ICSRs: comparative ROR analysis at the Preferred Term level for ceftazidime/avibactam versus ceftolozane/tazobactam; Table S5: Comparison of Preferred Terms Reported in EudraVigilance with Adverse Reactions Listed in the European Summary of Product Characteristics for Ceftazidime/Avibactam and Ceftolozane/Tazobactam.
