# Supplementary material for: Post-Marketing Reporting Patterns of Ceftazidime–Avibactam and Ceftolozane–Tazobactam: Analysis of Adverse Event Reports from EudraVigilance
Source: Antibiotics (Basel). 2026 Oct 5;15(10):982. doi: 10.3390/antibiotics15100982 (PMC13700556; doi:10.3390/antibiotics15100982)
Supplement: Supplementary file 1 [file antibiotics-15-00982-s001.zip › antibiotics-4525093-supplementary.pdf]

Table S1. Age-Stratified Comparative ROR Analysis at the System Organ Class Level for Ceftazidime/Avibactam versus Ceftolozane/Tazobactam

SOC	CA ICSRs in older patients	CT ICSRs in older patients	ROR [95% CI] in older patients	CA ICSRs in adult patients	CT ICSRs in adult patients	ROR [95% CI] in adult patients
Blood and lymphatic system disorders	45	13	1.10 [0.57, 2.11]	42	29	0.56 [0.33, 0.94]
Cardiac disorders	15	4	1.19 [0.39, 3.64]	13	9	0.59 [0.25, 1.42]
Ear and labyrinth disorders	3	1	0.94 [0.10, 9.14]	0	0	NE
Eye disorders	5	1	1.58 [0.18, 13.64]	8	1	3.42 [0.42, 27.61]
Gastrointestinal disorders	39	8	1.59 [0.72, 3.49]	29	11	1.12 [0.54, 2.30]
General disorders and administration site conditions	74	40	0.49 [0.32, 0.77]	76	40	0.74 [0.48, 1.16]
Hepatobiliary disorders	34	6	1.85 [0.76, 4.51]	35	11	1.38 [0.68, 2.79]
Immune system disorders	8	3	0.84 [0.22, 3.20]	12	4	1.27 [0.40, 4.01]
Infections and infestations	44	21	0.62 [0.35, 1.09]	58	31	0.75 [0.46, 1.21]
Injury, poisoning and procedural complications	59	33	0.49 [0.30, 0.79]	40	43	0.31 [0.19, 0.51]
Investigations	115	18	2.38 [1.39, 4.09]	96	13	3.89 [2.10, 7.20]
Metabolism and nutrition disorders	9	6	0.46 [0.16, 1.32]	7	9	0.31 [0.11, 0.86]
Musculoskeletal and connective tissue disorders	0	0	NE	5	6	0.34 [0.10, 1.14]
Neoplasms benign, malignant and unspecified (incl cysts and polyps)	0	0	NE	5	1	2.12 [0.25, 18.31]
Nervous system disorders	75	14	1.83 [1.00, 3.36]	52	21	1.05 [0.61, 1.81]
Psychiatric disorders	16	1	5.19 [0.68, 39.51]	20	3	2.91 [0.85, 9.96]
Renal and urinary disorders	66	17	1.26 [0.71, 2.24]	25	13	0.79 [0.40, 1.60]
Respiratory, thoracic and mediastinal disorders	18	5	1.14 [0.41, 3.13]	15	16	0.37 [0.18, 0.77]

Skin and subcutaneous tissue disorders	77	22	1.12 [0.67, 1.89]	72	23	1.40 [0.84, 2.34]
Surgical and medical procedures	0	0	NE	3	9	0.13 [0.04, 0.50]
Vascular disorders	14	9	0.47 [0.20, 1.12]	13	4	1.38 [0.44, 4.31]

Note: Adult patients were defined as those aged 18–64 years, whereas older patients were defined as those aged ≥65 years. CA, ceftazidime/avibactam; CT, ceftolozane/tazobactam; ICSR, Individual Case Safety Report; ROR, Reporting Odds Ratio; CI, confidence interval; NE, not estimable because no event was reported for either drug within the corresponding age stratum.

Table S2. Age-Stratified Comparative ROR Analysis at the Preferred Term Level for Ceftazidime/Avibactam versus Ceftolozane/Tazobactam.

Preferred Term (PT)	CA ICSRs in older patients	CT ICSRs in older patients	ROR [95% CI] in older patients	CA ICSRs in adult patients	CT ICSRs in adult patients	ROR [95% CI] in adult patients
Anaemia	5	2	0.78 [0.15, 4.09]	0	0	NE
Eosinophilia	0	0	NE	6	4	0.62 [0.17, 2.25]
Myelosuppression	0	0	NE	3	1	1.26 [0.13, 12.26]
Neutropenia	0	0	NE	6	8	0.30 [0.10, 0.89]
Leukopenia	4	3	0.41 [0.09, 1.87]	0	0	NE
Thrombocytopenia	15	3	1.59 [0.45, 5.59]	10	9	0.45 [0.18, 1.14]
Diarrhoea	19	1	6.21 [0.82, 46.82]	10	1	4.30 [0.55, 33.91]
Gastrointestinal haemorrhage	0	0	NE	5	1	2.12 [0.25, 18.31]
Melaena	3	1	0.94 [0.10, 9.14]	0	0	NE
Nausea	0	0	NE	3	1	1.26 [0.13, 12.26]
Vomiting	0	0	NE	4	1	1.69 [0.19, 15.26]
Condition aggravated	8	2	1.26 [0.26, 6.02]	9	1	3.86 [0.48, 30.75]
Death	3	11	0.08 [0.02, 0.29]	3	10	0.12 [0.03, 0.44]
Drug ineffective	19	5	1.20 [0.44, 3.29]	17	5	1.45 [0.53, 4.01]
Drug resistance	8	2	1.26 [0.26, 6.02]	5	5	0.41 [0.12, 1.45]
Multiple organ dysfunction syndrome	5	3	0.52 [0.12, 2.20]	5	3	0.70 [0.16, 2.96]
Pyrexia	13	2	2.08 [0.46, 9.33]	14	7	0.84 [0.33, 2.11]
Therapeutic product effect incomplete	0	0	NE	4	2	0.84 [0.15, 4.64]
Cholestasis	3	2	0.47 [0.08, 2.83]	6	3	0.84 [0.21, 3.40]
Hepatic cytolysis	5	1	1.58 [0.18, 13.64]	0	0	NE
Jaundice	0	0	NE	3	1	1.26 [0.13, 12.26]
Liver injury	0	0	NE	8	1	3.42 [0.42, 27.61]
Drug hypersensitivity	4	1	1.26 [0.14, 11.38]	8	2	1.70 [0.36, 8.10]
Clostridium difficile infection	4	1	1.26 [0.14, 11.38]	3	3	0.42 [0.08, 2.08]
Infection	4	1	1.26 [0.14, 11.38]	0	0	NE
Pathogen resistance	7	4	0.54 [0.16, 1.88]	13	6	0.91 [0.34, 2.44]
Pneumonia	3	2	0.47 [0.08, 2.83]	0	0	NE
Pseudomonas infection	0	0	NE	4	3	0.56 [0.12, 2.52]
Sepsis	5	4	0.39 [0.10, 1.46]	8	4	0.84 [0.25, 2.83]
Septic shock	5	2	0.78 [0.15, 4.09]	9	4	0.95 [0.29, 3.12]
Urinary tract infection	4	1	1.26 [0.14, 11.38]	0	0	NE
Off label use	49	11	1.45 [0.73, 2.88]	30	17	0.72 [0.38, 1.35]
Overdose	3	2	0.47 [0.08, 2.83]	0	0	NE

Product use in unapproved indication	3	13	0.07 [0.02, 0.24]	0	0	NE
Product use issue	8	2	1.26 [0.26, 6.02]	5	4	0.52 [0.14, 1.96]
C-reactive protein increased	3	3	0.31 [0.06, 1.55]	0	0	NE
Drug level increased	5	1	1.58 [0.18, 13.64]	0	0	NE
Platelet count decreased	34	1	11.54 [1.56, 85.09]	0	0	NE
Transaminases increased	8	1	2.55 [0.32, 20.54]	8	2	1.70 [0.36, 8.10]
White blood cell count decreased	11	1	3.53 [0.45, 27.56]	0	0	NE
White blood cell count increased	3	1	0.94 [0.10, 9.14]	0	0	NE
Coma	0	0	NE	10	1	4.30 [0.55, 33.91]
Depressed level of consciousness	0	0	NE	4	1	1.69 [0.19, 15.26]
Encephalopathy	13	2	2.08 [0.46, 9.33]	10	1	4.30 [0.55, 33.91]
Epilepsy	9	1	2.87 [0.36, 22.87]	3	1	1.26 [0.13, 12.26]
Seizure	13	1	4.19 [0.54, 32.30]	11	5	0.92 [0.32, 2.71]
Status epilepticus	3	1	0.94 [0.10, 9.14]	0	0	NE
Tremor	7	1	2.22 [0.27, 18.22]	4	1	1.69 [0.19, 15.26]
Confusional state	3	1	0.94 [0.10, 9.14]	0	0	NE
Delirium	0	0	NE	7	1	2.99 [0.36, 24.48]
Acute kidney injury	14	5	0.88 [0.31, 2.48]	6	6	0.41 [0.13, 1.30]
Renal failure	15	6	0.78 [0.30, 2.05]	3	3	0.42 [0.08, 2.08]
Renal impairment	30	4	2.46 [0.85, 7.12]	11	3	1.56 [0.43, 5.68]
Dyspnoea	8	1	2.55 [0.32, 20.54]	0	0	NE
Respiratory failure	3	1	0.94 [0.10, 9.14]	3	4	0.31 [0.07, 1.40]
Drug eruption	5	1	1.58 [0.18, 13.64]	0	0	NE
Erythema	14	2	2.24 [0.50, 10.00]	15	7	0.90 [0.36, 2.25]
Pruritus	7	5	0.43 [0.13, 1.38]	11	4	1.16 [0.36, 3.71]
Rash	25	7	1.13 [0.48, 2.68]	20	3	2.91 [0.85, 9.96]
Rash maculo-papular	8	1	2.55 [0.32, 20.54]	10	3	1.41 [0.38, 5.22]
Rash pruritic	3	1	0.94 [0.10, 9.14]	0	0	NE
Urticaria	3	4	0.23 [0.05, 1.04]	7	2	1.48 [0.30, 7.22]
Flushing	3	2	0.47 [0.08, 2.83]	0	0	NE
Hypotension	4	1	1.26 [0.14, 11.38]	6	1	2.55 [0.30, 21.38]

Note: Adult patients were defined as those aged 18–64 years, whereas older patients were defined as those aged ≥65 years. CA, ceftazidime/avibactam; CT, ceftolozane/tazobactam; ICSR, Individual Case

Safety Report; PT, Preferred Term; ROR, Reporting Odds Ratio; CI, confidence interval; NE, not estimable because no event was reported for either drug within the corresponding age stratum.

Table S3. Sensitivity analysis restricted to serious ICSRs: comparative ROR analysis at the System Organ Class level for ceftazidime/avibactam versus ceftolozane/tazobactam.

SOC	CA serious ICSRs	CT serious ICSRs	ROR [95% CI]
Blood and lymphatic system disorders	88	49	0.69 [0.47–1.00]
Cardiac disorders	32	19	0.66 [0.37–1.18]
Congenital, familial and genetic disorders	2	2	0.40 [0.06–2.84]
Ear and labyrinth disorders	5	2	1.00 [0.19–5.18]
Eye disorders	14	2	2.83 [0.64–12.51]
Gastrointestinal disorders	73	27	1.09 [0.69–1.72]
General disorders and administration site conditions	212	119	0.62 [0.47–0.81]
Hepatobiliary disorders	74	17	1.81 [1.05–3.11]
Immune system disorders	21	7	1.20 [0.51–2.86]
Infections and infestations	146	91	0.57 [0.42–0.77]
Injury, poisoning and procedural complications	139	103	0.45 [0.34–0.61]
Investigations	226	37	2.94 [2.03–4.27]
Metabolism and nutrition disorders	21	16	0.51 [0.26–1.00]
Musculoskeletal and connective tissue disorders	13	9	0.57 [0.24–1.35]
Neoplasms benign, malignant and unspecified (incl cysts and polyps)	12	6	0.80 [0.30–2.14]
Nervous system disorders	146	41	1.51 [1.04–2.19]
Product issues	2	8	0.10 [0.02–0.46]
Psychiatric disorders	40	4	4.14 [1.47–11.67]
Renal and urinary disorders	101	43	0.93 [0.64–1.36]
Respiratory, thoracic and mediastinal disorders	35	24	0.57 [0.33–0.97]
Skin and subcutaneous tissue disorders	109	28	1.64 [1.06–2.53]
Surgical and medical procedures	6	19	0.12 [0.05–0.30]
Vascular disorders	28	16	0.69 [0.37–1.29]

Table S4. Sensitivity analysis restricted to serious ICSRs: comparative ROR analysis at the Preferred Term level for ceftazidime/avibactam versus ceftolozane/tazobactam.

Preferred Term (PT)	CA serious ICSRs	CT serious ICSRs	ROR [95% CI]
Abdominal pain	2	2	0.40 [0.06–2.84]
Acute kidney injury	23	10	0.92 [0.43–1.95]
Acute respiratory distress syndrome	2	1	0.80 [0.07–8.85]
Agranulocytosis	8	7	0.45 [0.16–1.26]
Altered state of consciousness	8	2	1.61 [0.34–7.60]
Anaemia	6	6	0.40 [0.13–1.24]
Anaphylactic reaction	3	3	0.40 [0.08–1.98]
Angioedema	2	2	0.40 [0.06–2.84]
Anuria	2	1	0.80 [0.07–8.85]
Ascites	2	1	0.80 [0.07–8.85]
Aspergillus infection	1	2	0.20 [0.02–2.20]
Asthenia	3	1	1.20 [0.12–11.58]
Atrial fibrillation	2	5	0.16 [0.03–0.82]
Bacteraemia	1	4	0.10 [0.01–0.89]
Blood alkaline phosphatase increased	3	2	0.60 [0.10–3.60]
Blood creatinine increased	31	1	12.82 [1.74–94.25]
Blood sodium decreased	3	1	1.20 [0.12–11.58]
Blood urea increased	2	1	0.80 [0.07–8.85]
Bone pain	2	1	0.80 [0.07–8.85]
C-reactive protein increased	6	3	0.80 [0.20–3.21]
Candida infection	2	1	0.80 [0.07–8.85]
Cardiac arrest	3	3	0.40 [0.08–1.98]
Cardiac disorder	2	1	0.80 [0.07–8.85]
Cardio-respiratory arrest	2	3	0.26 [0.04–1.59]
Cardiogenic shock	2	1	0.80 [0.07–8.85]
Cerebrovascular accident	2	1	0.80 [0.07–8.85]
Chest pain	2	2	0.40 [0.06–2.84]
Cholestasis	10	4	1.00 [0.31–3.21]
Chronic kidney disease	2	1	0.80 [0.07–8.85]
Clostridium difficile colitis	4	8	0.20 [0.06–0.66]
Clostridium difficile infection	4	3	0.53 [0.12–2.39]
Coagulopathy	6	1	2.41 [0.29–20.09]
Coma	14	1	5.67 [0.74–43.32]
Condition aggravated	19	4	1.92 [0.65–5.68]
Confusional state	5	1	2.01 [0.23–17.23]
Death	20	49	0.14 [0.08–0.25]
Delirium	15	2	3.03 [0.69–13.34]
Depressed level of consciousness	13	1	5.26 [0.69–40.39]
Dermatitis allergic	2	1	0.80 [0.07–8.85]
Dermatitis exfoliative generalised	2	1	0.80 [0.07–8.85]
Diarrhoea	30	5	2.45 [0.94–6.37]
Dizziness	4	1	1.60 [0.18–14.39]
Drug eruption	8	1	3.22 [0.40–25.84]
Drug hypersensitivity	10	2	2.01 [0.44–9.23]
Drug ineffective	55	15	1.50 [0.83–2.69]
Drug ineffective for unapproved indication	2	5	0.16 [0.03–0.82]

Drug interaction	3	1	1.20 [0.12–11.58]
Drug level increased	5	1	2.01 [0.23–17.23]
Drug reaction with eosinophilia and systemic symptoms	4	2	0.80 [0.15–4.38]
Drug resistance	25	5	2.03 [0.77–5.34]
Dyspnoea	8	3	1.07 [0.28–4.05]
Electrolyte imbalance	2	1	0.80 [0.07–8.85]
Encephalopathy	27	3	3.68 [1.11–12.22]
Enterococcal infection	2	1	0.80 [0.07–8.85]
Eosinophil count increased	3	1	1.20 [0.12–11.58]
Eosinophilia	7	1	2.81 [0.34–22.96]
Epilepsy	12	2	2.42 [0.54–10.87]
Erythema	20	6	1.34 [0.53–3.37]
Face oedema	5	1	2.01 [0.23–17.23]
Flushing	5	2	1.00 [0.19–5.18]
Fungal infection	4	1	1.60 [0.18–14.39]
Gamma-glutamyltransferase increased	3	1	1.20 [0.12–11.58]
Gastrointestinal haemorrhage	7	4	0.70 [0.20–2.40]
General physical health deterioration	3	3	0.40 [0.08–1.98]
Generalised tonic-clonic seizure	4	3	0.53 [0.12–2.39]
Haematochezia	3	1	1.20 [0.12–11.58]
Haemorrhage	2	2	0.40 [0.06–2.84]
Headache	5	1	2.01 [0.23–17.23]
Hepatic cytolysis	7	4	0.70 [0.20–2.40]
Hepatic failure	2	1	0.80 [0.07–8.85]
Hyperhidrosis	2	1	0.80 [0.07–8.85]
Hyperkalaemia	2	2	0.40 [0.06–2.84]
Hypernatraemia	7	1	2.81 [0.34–22.96]
Hypersensitivity	4	1	1.60 [0.18–14.39]
Hypokalaemia	4	6	0.26 [0.07–0.94]
Hypotension	11	6	0.73 [0.27–1.99]
Incorrect dose administered	5	6	0.33 [0.10–1.09]
Incorrect product administration duration	1	4	0.10 [0.01–0.89]
Infection	9	3	1.20 [0.32–4.47]
Jaundice	3	1	1.20 [0.12–11.58]
Klebsiella infection	7	1	2.81 [0.34–22.96]
Leukopenia	5	7	0.28 [0.09–0.89]
Liver function test abnormal	2	1	0.80 [0.07–8.85]
Liver injury	10	1	4.03 [0.51–31.64]
Loss of consciousness	3	1	1.20 [0.12–11.58]
Malaise	4	1	1.60 [0.18–14.39]
Melaena	5	1	2.01 [0.23–17.23]
Metabolic acidosis	2	2	0.40 [0.06–2.84]
Multiple organ dysfunction syndrome	15	6	1.00 [0.38–2.60]
Multiple-drug resistance	5	2	1.00 [0.19–5.18]
Muscle twitching	4	1	1.60 [0.18–14.39]
Myelosuppression	9	1	3.63 [0.46–28.73]
Myocardial infarction	2	1	0.80 [0.07–8.85]
Myoclonus	10	1	4.03 [0.51–31.64]
Nausea	4	1	1.60 [0.18–14.39]
Nephropathy toxic	2	2	0.40 [0.06–2.84]

Neurological symptom	2	1	0.80 [0.07–8.85]
Neurotoxicity	3	2	0.60 [0.10–3.60]
Neutropenia	8	8	0.39 [0.15–1.06]
Off label use	104	37	1.14 [0.77–1.70]
Organ failure	1	2	0.20 [0.02–2.20]
Osteomyelitis	3	3	0.40 [0.08–1.98]
Overdose	4	5	0.32 [0.08–1.19]
Oxygen saturation decreased	7	1	2.81 [0.34–22.96]
Pain	4	1	1.60 [0.18–14.39]
Pancytopenia	3	5	0.24 [0.06–1.00]
Pathogen resistance	36	28	0.49 [0.30–0.82]
Platelet count decreased	51	1	21.59 [2.97–156.87]
Platelet count increased	3	1	1.20 [0.12–11.58]
Pneumonia	10	9	0.44 [0.18–1.09]
Pneumonia pseudomonal	1	4	0.10 [0.01–0.89]
Postoperative wound infection	1	2	0.20 [0.02–2.20]
Product administered to patient of inappropriate age	3	1	1.20 [0.12–11.58]
Product prescribing error	2	2	0.40 [0.06–2.84]
Product use in unapproved indication	7	39	0.06 [0.03–0.15]
Product use issue	26	10	1.04 [0.50–2.18]
Pruritus	18	4	1.82 [0.61–5.41]
Pseudomonas infection	8	15	0.21 [0.09–0.49]
Pseudomonas test positive	2	1	0.80 [0.07–8.85]
Purpura	3	1	1.20 [0.12–11.58]
Pyrexia	26	8	1.31 [0.59–2.92]
Rash	36	8	1.83 [0.84–3.99]
Rash maculo-papular	11	3	1.47 [0.41–5.31]
Red blood cell count decreased	3	1	1.20 [0.12–11.58]
Renal failure	20	14	0.56 [0.28–1.12]
Renal impairment	41	14	1.18 [0.63–2.19]
Respiratory failure	7	7	0.40 [0.14–1.14]
Seizure	29	11	1.06 [0.52–2.14]
Sepsis	14	8	0.70 [0.29–1.67]
Septic shock	19	5	1.53 [0.57–4.13]
Shock haemorrhagic	2	1	0.80 [0.07–8.85]
Status epilepticus	4	1	1.60 [0.18–14.39]
Stevens-Johnson syndrome	2	1	0.80 [0.07–8.85]
Systemic candida	1	2	0.20 [0.02–2.20]
Systemic inflammatory response syndrome	2	1	0.80 [0.07–8.85]
Tachycardia	3	1	1.20 [0.12–11.58]
Therapeutic product effect incomplete	5	1	2.01 [0.23–17.23]
Therapy non-responder	2	1	0.80 [0.07–8.85]
Thrombocytopenia	26	16	0.64 [0.34–1.21]
Tongue oedema	1	2	0.20 [0.02–2.20]
Transaminases increased	15	1	6.09 [0.80–46.26]
Treatment failure	2	3	0.26 [0.04–1.59]
Tremor	11	2	2.22 [0.49–10.05]
Underdose	2	2	0.40 [0.06–2.84]
Upper gastrointestinal haemorrhage	2	1	0.80 [0.07–8.85]

Urinary tract infection	6	2	1.20 [0.24–5.98]
Urticaria	2	3	0.26 [0.04–1.59]
Ventricular tachycardia	2	1	0.80 [0.07–8.85]
Vomiting	6	2	1.20 [0.24–5.98]
Weight decreased	1	2	0.20 [0.02–2.20]
White blood cell count decreased	20	2	4.07 [0.95–17.50]
White blood cell count increased	5	3	0.66 [0.16–2.80]

Table S5. Comparison of Preferred Terms Reported in EudraVigilance with Adverse Reactions Listed in the European Summary of Product Characteristics for Ceftazidime/Avibactam and Ceftolozane/Tazobactam.

Preferred Term (PT)	CA, n	CT, n	SmPC CA	SmPC CT
Acute kidney injury	23	12	Yes	No
Adverse event	0	17	No	No
Agranulocytosis	8	7	Yes	No
Alanine aminotransferase increased	8	0	Yes	Yes
Altered state of consciousness	8	2	No	No
Anaemia	6	6	No	Yes
Anaphylactic reaction	3	3	Yes	No
Anaphylactic shock	4	0	No	No
Antibiotic level above therapeutic	3	0	No	No
Aspartate aminotransferase increased	7	0	Yes	Yes
Asthenia	3	2	No	No
Atrial fibrillation	2	5	No	Yes
Bacteraemia	1	4	No	No
Bacterial infection	3	0	No	No
Bilirubin conjugated increased	4	0	No	No
Blood alkaline phosphatase increased	3	2	Yes	Yes
Blood bilirubin increased	17	0	No	No
Blood creatinine increased	31	2	Yes	No
Blood potassium decreased	3	0	No	Yes – corresponding ADR: Hypokalaemia
Blood pressure decreased	4	0	No	No
Blood pressure increased	4	0	No	No
Blood sodium decreased	3	1	No	No
Blood sodium increased	3	0	No	No
Cardiac arrest	3	3	No	No
Cardiac failure	3	0	No	No
Cardiac failure acute	3	0	No	No
Cardio-respiratory arrest	2	3	No	No
Chest discomfort	3	0	No	No
Chills	5	0	No	No
Cholestasis	11	5	No	No
Clostridium difficile colitis	4	8	Yes – corresponding ADR: Clostridioides difficile colitis	Yes – corresponding ADR: Clostridioides difficile colitis
Clostridium difficile infection	4	3	No	Yes – corresponding ADR: Clostridioides difficile infection
Coagulopathy	6	1	No	No

Coma	14	1	No – mentioned in §§4.4/4.9, not listed as ADR in §4.8	No
Condition aggravated	22	4	No	No
Confusional state	5	2	No	No
Coombs direct test positive	3	0	Yes	Yes – corresponding ADR: Coombs test positive
Coombs test positive	3	0	Yes – corresponding ADR: Coombs direct test positive	Yes
C-reactive protein decreased	3	0	No	No
C-reactive protein increased	7	5	No	No
Death	20	49	No	No
Decubitus ulcer	3	0	No	No
Delirium	15	2	No	No
Depressed level of consciousness	13	1	No	No
Dermatitis allergic	2	3	No	No
Diarrhoea	30	5	Yes	Yes
Disease progression	10	0	No	No
Disease recurrence	4	0	No	No
Disturbance in attention	3	1	No	No
Dizziness	4	2	Yes	Yes
Drug effective for unapproved indication	6	0	No	No
Drug eruption	9	1	No	No
Drug hypersensitivity	13	3	No	No
Drug ineffective	56	18	No	No
Drug ineffective for unapproved indication	2	7	No	No
Drug interaction	4	1	No	No
Drug level increased	6	1	No	No
Drug reaction with eosinophilia and systemic symptoms	4	2	Yes	No
Drug resistance	25	12	No	No
Drug-induced liver injury	16	0	No	No
Dysbiosis	13	0	No	No
Dysphoria	4	0	No	No
Dyspnoea	11	5	No	Yes
Encephalopathy	27	3	No – mentioned in §§4.4/4.9, not listed as ADR in §4.8	No
Eosinophil count increased	3	1	No	No
Eosinophilia	14	4	Yes	No
Epilepsy	12	2	No	No
Erythema	31	11	No	No
Eyelid oedema	3	0	No	No
Face oedema	5	1	No	No

Fatigue	4	0	No	No
Flushing	5	3	No	No
Full blood count decreased	3	0	No	No
Fungal infection	4	1	No	No
Gamma-glutamyltransferase increased	4	1	Yes	Yes
Gastrointestinal haemorrhage	7	4	No	No
General physical health deterioration	3	3	No	No
Generalised tonic-clonic seizure	4	3	No	No
Granulocyte count decreased	3	0	No	No
Haematochezia	3	1	No	No
Haemodynamic instability	3	0	No	No
Haemoglobin decreased	9	0	No	No
Haemolysis	3	0	No	No
Haemolytic anaemia	4	0	Yes	No
Hallucination, visual	3	1	No	No
Headache	5	3	Yes	Yes
Heart rate increased	10	0	No	No
Hepatic cytolysis	8	4	No	No
Hepatic enzyme increased	16	1	No	No
Hepatic function abnormal	23	0	No	No
Hyperbilirubinaemia	0	4	No	No
Hypernatraemia	7	3	No	No
Hyperpyrexia	4	1	No	No
Hypersensitivity	4	2	No	No
Hypokalaemia	4	6	No	Yes
Hypotension	11	6	No	Yes
Hypoxia	4	0	No	No
Incorrect dose administered	5	8	No	No
Incorrect drug administration rate	3	0	No	No
Incorrect product administration duration	3	5	No	No
Indifference	3	0	No	No
Infection	9	4	No	No
Infusion site phlebitis	0	3	Yes	Yes – corresponding ADR: Phlebitis / infusion-site reactions
Jaundice	4	1	Yes	No
Klebsiella infection	7	1	No	No
Leukopenia	5	7	Yes	No
Liver injury	10	1	No	No
Loss of consciousness	3	1	No	No
Lower gastrointestinal haemorrhage	3	0	No	No
Lung infiltration	0	3	No	No
Macule	3	0	No	No
Malaise	4	2	No	No

Melaena	5	1	No	No
Mental disorder	4	0	No	No
Mixed liver injury	3	0	No	No
Multiple organ dysfunction syndrome	15	6	No	No
Multiple-drug resistance	5	2	No	No
Muscle twitching	4	1	No	No
Myelosuppression	9	1	No	No
Myocardial ischaemia	3	0	No	No
Myoclonus	10	3	No – mentioned in §4.4, not listed as ADR in §4.8	No
Nausea	6	1	Yes	Yes
Nephropathy toxic	2	4	No	No
Nervous system disorder	4	0	No	No
Neurotoxicity	3	2	No	No
Neutropenia	8	8	Yes	Yes
Neutrophil count decreased	6	0	Yes – corresponding ADR: Neutropenia	Yes – corresponding ADR: Neutropenia
Neutrophil percentage increased	3	0	No	No
No adverse event	1	5	No	No
Oedema peripheral	4	0	No	No
Off label use	111	56	No	No
Oliguria	3	0	No	No
Osteomyelitis	3	3	No	No
Overdose	4	6	No	No
Oxygen saturation decreased	7	1	No	No
Pain	4	2	No	No
Pancytopenia	3	5	No	No
Pathogen resistance	37	29	No	No
Petechiae	3	1	No	No
Platelet count decreased	55	1	Yes – corresponding ADR: Thrombocytopenia	No
Platelet count increased	3	1	Yes – corresponding ADR: Thrombocytosis	Yes – corresponding ADR: Thrombocytosis
Pneumonia	10	9	No	No
Pneumonia klebsiella	5	0	No	No
Pneumonia pseudomonal	1	4	No	No
Procalcitonin increased	5	0	No	No
Product administered to patient of inappropriate age	3	1	No	No
Product storage error	0	3	No	No
Product use in unapproved indication	9	48	No	No
Product use issue	28	12	No	No
Pruritus	23	10	Yes	No
Pseudomonas infection	8	15	No	No
Purpura	3	1	No	No

Pyrexia	29	11	Yes	Yes
Rash	50	15	No	Yes
Rash erythematous	14	0	No	No
Rash maculo-papular	18	4	Yes	No
Rash pruritic	5	1	No	No
Recalled product administered	4	0	No	No
Red blood cell count decreased	4	1	No	No
Renal failure	21	14	No	Yes
Renal impairment	41	14	No	Yes
Renal injury	6	0	No	No
Respiratory failure	7	7	No	No
Respiratory rate increased	3	0	No	No
Seizure	29	11	No – convulsion mentioned in §§4.4/4.9, not listed as ADR in §4.8	No
Sepsis	14	8	No	No
Septic shock	19	6	No	No
Somnolence	5	0	No	No
Status epilepticus	4	1	No – non-convulsive status epilepticus mentioned in §4.4, not listed as ADR in §4.8	No
Surgery	0	4	No	No
Syncope	3	0	No	No
Tachycardia	4	2	No	Yes
Tachypnoea	7	0	No	No
Temperature intolerance	3	0	No	No
Therapeutic product effect incomplete	5	3	No	No
Thrombocytopenia	28	16	Yes	No
Toxic encephalopathy	4	0	No	No
Toxic skin eruption	3	1	No	No
Transaminases increased	16	3	No	Yes
Treatment failure	2	6	No	No
Tremor	11	2	No – mentioned in §4.4, not listed as ADR in §4.8	No
Urinary tract infection	6	2	No	No
Urticaria	11	6	Yes	Yes
Vascular device infection	3	0	No	No
Vomiting	6	2	Yes	Yes
White blood cell count decreased	21	2	Yes – corresponding ADR: Leukopenia	No
White blood cell count increased	5	3	No	No

Preferred Terms reported in at least three ICSRs for either CA or CT were included in the comparison with the respective European Summary of Product Characteristics (SmPC). A PT was considered listed

when the same term or a clinically corresponding adverse reaction was reported in the SmPC. CA, ceftazidime/avibactam; CT, ceftolozane/tazobactam.
